# Supercritical CO_2_ Extraction of Bioactive Compounds from Mango (*Mangifera indica* L.) Peel and Pulp

**DOI:** 10.3390/foods10092201

**Published:** 2021-09-17

**Authors:** José Villacís-Chiriboga, Stefan Voorspoels, Maarten Uyttebroek, Jenny Ruales, John Van Camp, Edwin Vera, Kathy Elst

**Affiliations:** 1 Flemish Institute for Technological Research (VITO), Business Unit Separation and Conversion Technology, Boeretang 200, 2400 Mol, Belgium; jose.villacis-chiriboga@vito.be (J.V.-C.); stefan.voorspoels@vito.be (S.V.); Maarten.Uyttebroek@flandersfood.com (M.U.); 2 Department of Food Technology, Safety and Health, Ghent University, Coupure Links 653, 9000 Ghent, Belgium; John.VanCamp@UGent.be; 3 Department of Food Science and Biotechnology, Escuela Politécnica Nacional, Ecuador, Campus Rubén Orellana, Ladrón de Guevara E11-253, Quito P.O. Box 17 012759, Ecuador; jenny.ruales@epn.edu.ec (J.R.); edwin.vera@epn.edu.ec (E.V.)

**Keywords:** carotenoids, phenolics, antioxidant, mango, supercritical CO_2_, response surface optimization

## Abstract

The potential of supercritical CO_2_ (SC-CO_2_) for the extraction of bioactive compounds from mango by-products was assessed. Carotenoid extraction was optimized using a design of experiments based on temperature (35, 55 and 70 °C), pressure (10 and 35 MPa) and co-solvent addition (0%, 10% and 20% of ethanol or acetone). Moreover, the co-extraction of phenolic acids, flavonoids and xanthonoids was evaluated in a subset of parameters. Finally, a comparison was made between SC-CO_2_ and a two-step organic solvent extraction of the bioactive compounds from the pulp and peel fractions of two Ecuadorian varieties. The optimal extraction temperature was found to be dependent on the bioactive type, with phenolics requiring higher temperature than carotenoids. The optimal overall conditions, focused on maximal carotenoids recovery, were found to be 55 °C, 35 MPa and 20% of ethanol. The main carotenoid was β-carotene, while phenolics differed among the varieties. The bioactive content of the peel was up to 4.1-fold higher than in the pulp fraction. Higher antioxidant activity was found in the extracts obtained with organic solvents. SC-CO_2_ is a promising technology for the isolation of valuable compounds from mango by-products.

## 1. Introduction

Mango (*Mangifera indica* L.) is among the most traded tropical fruits in the world with around 48 thousand tons produced in 2017. Nevertheless, large amounts of peels (7–24%) and stones (45–85%) are discarded after industrial processing of the fruit. Such by-products represent a significant material source of compounds with potentially high commercial value and applicability for various purposes. In this line, the concept of biorefinery aims to produce valuable chemicals, food, feed and energy by using biomass as feedstock through various transformation steps. More specifically for mango, several studies have stated that its by-products could potentially be used for the extraction of macro molecules such as pectins, oils and starch, but also bioactive compounds such as carotenoids and phenolics [1].

Carotenoids and phenolic compounds are among the major bioactive compounds of mango. Studies have indicated the positive effects of carotenoids on human health, i.e., radical scavenging, cancer prevention, cardiovascular diseases, cataracts and neural tube defects [2]. Similarly, phenolic compounds exert various biological activities, including antimicrobial, anti-cardiovascular, anti-obesity, antioxidant, anti-hyperglycemic and anticancer activities [3]. Moreover, polyphenols of mango have been shown to exert chemopreventive and anti-inflammatory activities [4]. Such activities make of mango by-products a potential source of compounds with application in various industrial sectors.

Bioactive compounds are traditionally extracted from vegetable matrices using organic solvents, which can leave potentially toxic residues and be harmful when released into the environment. In contrast, supercritical CO_2_ (SC-CO_2_) provides a sustainable, environmentally friendly and cost-effective alternative solvent that offers the possibility to tailor the extraction process by changing its pressure or altering its polarity by the addition of low concentrations of organic solvents. It is especially appropriate for the isolation of lipophilic compounds. Moreover, it allows one to avoid thermal damage of labile bioactive compounds, given the low working temperature for reaching the supercritical state (T = 31.1 °C and P = 7.38 MPa) [5].

Given the crescent interest of SC-CO_2_ as an extraction solvent of carotenoids, in the last few years, several matrices have been extracted with this methodology, such as microalgae [6] and carrot peels [7]. Nevertheless, as far as mango is concerned, the few scientific reports available focus on the tree leaves as main sources of bioactive compounds. To the best of our knowledge, the research on the application of SC-CO_2_ is still limited. Garcia-Mendoza et al. (2015) [8], determined the total extraction yield of carotenoids, phenols and flavonoids from one variety under a single SC-CO_2_ extraction condition, followed by a pressurized EtOH extraction. In a similar study, Sánchez-Camargo et al. (2019) [9] optimized the conditions of SC-CO_2_ extraction of carotenoids from mango peels of the variety “Sugar”. The authors evaluated the total carotenoid content as a function of the drying techniques (freeze drying and air drying). Furthermore, the carotenoid extract obtained under the best working parameters was evaluated in terms of its carotenoids profile and as protector of the oxidation of sunflower oil. Since both carotenoids and polyphenols are composed of different molecules with different polarity and weight, it is expected that the extraction yield of individual compounds will depend on the conditions applied. Given the fact that the individual molecules may vary among the different mango fractions and varieties as well as the storage/processing conditions, it is important to understand their individual behavior as a function of the conditions used for extraction. For this purpose, response surface methodology (RSM) provides a powerful statistical tool to model and predict the outcome of an extraction in function of the conditions applied based on a limited amount of experiments [10].

The present work aims to investigate the use of SC-CO_2_ for the extraction of bioactive compounds from the peel and pulp of mango. To obtain a better knowledge on the effect of temperature, pressure and co-solvent on the extraction of carotenoids, the extraction of the individual carotenoids is analyzed and modelled by the RSM methodology. Moreover, the extracts of a subset of the most promising extraction conditions are characterized in detail for their composition in the individual phenolics. Based on the modelling results, optimized conditions are proposed and used for the supercritical extraction of bioactive compounds from the pulp and peel of different mango varieties. The results are compared to a two-step organic solvent extraction.

## 2. Materials and Methods

### 2.1. Sample Preparation

In this study, two batches of mango were selected. For the first batch, the dejuiced whole fruit, with no discrimination between the pulp and peel, was used in order to have a maximal amount of a representative mixed material required for the optimization process. The variety “Kasturi”, grown in Israel, was purchased on a local market in Belgium. The selected mangoes, which were commercially ripe, similar in size and free of mechanical or biological damages, were immediately taken to the laboratory and kept at 8 °C. The mangoes were manually washed, and the seeds were manually removed from the fruits. The remaining pulp (entire pulp after seed removal) and peel fraction was juiced with an Angel Juicer 8500s (Angelia, Sasang-gu, Busan, Korea) and the juice and pulp were frozen with liquid nitrogen, freeze dried, milled and sieved to reject the material with a diameter greater than 500 μm. The average particle size was 496 ± 71 μm (weight basis), measured by laser diffraction. The samples were then stored in vacuum bags away from light and kept at −20 °C until further analysis.

The second type of samples was extracted at the optimal conditions and the samples were used to study local Ecuadorian varieties and the effect of the separation of pulp and peel. The varieties “Kent” and “Haden” were obtained from local growers in the province of Imbabura—Ecuador (0°27′06.4′′ N; 77°59′22.6′′ W). The mangoes were selected based on an appropriate maturity degree, uniform shape and size and no damages of biological or mechanical origin. In the laboratory, the mangoes were manually washed and the three fractions—peel, pulp and stones—were manually separated. The pulp was juiced with an industrial juicer machine and the pulp resulting from this step, along with the peel, was freeze dried, milled and the process continued as for the first batch of mango.

### 2.2. Extraction with Supercritical CO_2_

The tests were performed with a laboratory scale Jasco SFE system from JASCO Isogen Life Science [11].

For each extraction, 2.0 g of freeze-dried powder pulp was loaded in a 10 mL capacity extractor vessel, and the empty space was filled with glass wool. CO_2_ constantly flowed at 1 mL·min^−1^ at a pressure of 10 or 35 MPa, at a temperature of 35, 55 or 70 °C and with pure CO_2_ or a co-solvent percentage of 10% or 20% ethanol (EtOH); alternatively, 10% or 20% acetone was used. All the experiments were performed randomly and in duplicate. A total extraction time of 3 h was applied, but the extract was collected in different collection vials as a function of time (10 min per vial). Amber-colored glass collection vials were used to avoid light degradation of the extracted molecules. As explained above, a modifier solvent was added at the depressurization point to avoid precipitation. The same solvent was used as the co-solvent of the experiment, except that additionally butylhydroxytoluene BHT (0.1%) was added to preserve the extract until the analysis. The modifier flow was set to obtain a total solvent flow (co-solvent + modifier) of 0.5 mL·min^−1^ in order to always have the same volume of collected extract in the vials. To correct for the differences in BHT content in the final extract (as this was uniquely dosed through the modifier solvent), additional BHT was directly added to the collection vials, always to keep its final concentration at 0.1%. After the extraction, the solvents were evaporated with a N_2_ flux and the dry extracts were stored under −18 °C. For the samples that were used for measuring the antioxidant activity, no BHT was added to the vial nor to the modifier.

In between successive extractions, the tubing of the equipment was extensively rinsed with isopropanol to avoid any contamination for remnants.

### 2.3. Solvent Extraction

The solvents system used were previously optimized after performing tests with several organic solvents and their mixtures. Hence, two successive extractions were applied. A combination of acetone:methanol (70:30; *v*/*v*) was first applied, then an extraction with dichloromethane:methanol (50:50; *v*/*v*) was carried out in an ultrasound bath Branson 5510 (Branson ultrasonics corporation, Danbury, CT, USA) at 4 °C. Both solvent systems contained 0.1% BHT. The ratio of material to solvent was 1:10 (*w*/*v*), and the time was set at 45 min for each extraction. At the end, both extracts were mixed together and separated in aliquots. The solvents were evaporated and the dried extracts were stored at −18 °C. As explained above, the samples used for the antioxidant activity measurement were not stabilized with BHT.

### 2.4. Determination of Dry Matter Content

The amount of dry matter was assessed by weight difference. For this, 5.0 g of dry sample were dried in an oven for 24 h at 105 °C, the difference in mass was calculated and used for correcting the values obtained in the experiments.

### 2.5. Determination of the Total Carotenoid Content

One aliquot of 2 mL of the extract was dried and before the analysis it was dispersed in EtOH (95%). Then, the absorbance of an appropriately diluted sample was analyzed in a spectrophotometer (Tecan, Infinite, M200 Pro) using ethanol (95%) as a blank at 470, 649 and 664 nm. The total carotenoid concentrations were calculated in duplicate from the different absorbances using the equations of Lichtenthaler and Buschmann (2001) [12].

### 2.6. Determination of the Individual Carotenoids

The protocol was based on the work of Bijttebier et al. (2013) [13]. One µL of extract was injected on a 2.1 mm × 100 mm, 1.8 μm HSS C_18_ SB column (Waters, Milford, MA, USA) at 35 °C in a Waters Acquity UPLC^®^. Mobile phase A consisted of water +5 mM ammonium acetate:methanol:acetonitrile:ethylacetate (50/22.5/22.5/5 *v*/*v*/*v*/*v*) and mobile phase B consisted of acetonitrile:ethylacetate (50/50 *v*/*v*) with a flow rate of 500 μL·min^−1^. The following gradient (%/min B) was applied: 10/0; 10/0.1; 30/0.8; 91/20.0; 100/20.1; 100/20.4; 10/20.5; 10/23. For detection, an orbitrap mass spectrometer (Thermo Fisher Scientific, Waltham, MA, USA) operating with an APCI was used. A full scan of the ions was acquired at m/z range of 200 to 1400 at a resolution of 70,000 full width at half-maximum.

As the internal standard, 36.25 ng·mL^−1^ of trans-β-Apo-8′-carotenal was used. The concentration of the standards (α-carotene, antheraxanthin, β-cryptoxanthin, β-carotene, fucoxanthin, trans-lutein, trans-violaxanthin, zeaxanthin) ranged between 0.034 and 26.55 µg·mL^−1^ (average). In addition, 3 quality controls (corresponding to the standard 5) were placed in between the injections of the samples. The lowest calibration point was set as the limit of quantification. The relative standard deviation of these controls was on average 6.35 ± 4.21%.

### 2.7. Determination of Individual Phenolic Compounds

The analytical method was based on the work of De Paepe et al. (2013) [14]. Five µL of extract were injected on a 3.0 mm × 150 mm, 1.7 μm BEH SHIELD RP_18_ column (Waters, Milford, MA, USA) at 40 °C in a Waters Acquity UPLC^®^. Mobile phase A consisted of water + 0.1% formic acid and mobile phase B constituted acetonitrile +0.1% formic acid flowing at 500 μL·min^−1^ at the following gradient (%/min B): 0–26/9.91; 65/18.51; 100/18.76–25.76. An orbitrap mass spectrometer (Thermo Fisher Scientific) coupled with an ESI source was used for identification. All ions were identified in a m/z range of 70–10,500 at a mass resolving power of 70,000 full width at half-maximum.

Daidzein was included as internal standard in a concentration of 50 ng·mL^−1^ in all the extracts. The concentration of the standards (aromadendrin, astragalin, avicularin, caffeic acid, catechin, chlorogenic acid, cyanoroside, epicatechin, ferulic acid, gallic acid, hyperin, isoquercitrin, kaempferol, mangiferin, naringenin, *p*-coumaric acid, phlorizin, protocatechuic acid, quercetin, quercitrin, taxifolin) ranged between 3.19 and 9848.47 µg·L^−1^ (average). Additionally, 7 quality controls (corresponding to the standard 3) were placed in between the injections of the samples. The lowest calibration point was set as the limit of quantification. The relative standard deviation of these controls was on average 6.13 ± 1.48%.

### 2.8. Determination of Antioxidant Activity

To assess the antioxidant properties of the samples, the 2,2-diphenyl-1-picrylhydrazyl (DPPH·) test, which monitors the neutralization of the stable DPPH· radical, was carried out. Additionally, the FRAP method was applied. This method consists of measuring the increase in absorbance at 593 nm of the blue reaction when the TPTZ-Fe^+3^ complex is reduced to TPTZ-Fe^+2^.

The DPPH· method was performed in duplicate per extraction, following the procedure of Brand-Williams et al. (1995) [15] with modifications. To prepare the stock solution, 3.0 mg of DPPH· was dissolved to a final concentration of 20 mM. In a next step, the stock solution was diluted with MeOH to a concentration of 200 µM. The samples were prepared as follows. First, 2 mL of extract was taken, evaporated and re-dissolved with 2 mL dimethyl sulfoxide (DMSO). A series of at least 6 different aliquots was taken from this DMSO solution, with a volume ranging between 0.5 and 300 µL, which was adjusted in volume to 500 µL with MeOH. For each test, 100 µL of the DPPH· working solution and 100 µL of each aliquot were mixed in a 96-well plate. The DPPH· working solution mixed with MeOH and pure MeOH was used as the blank. The mixtures were allowed to react for 30 min away from light. The absorbance was taken at 517 nm. Results were expressed as IC_50_. The final value was related to the biomass intake during the extraction (in DM) and expressed as µg biomass on dry matter base per mL (µg·mL^−1^).

The FRAP method was done according to the modified method of Benzie and Strain (1996) [16]. To prepare the FRAP solution, buffer acetate of 300 mM, 2,4,6-Tris-(2-pyridyl)-s-triazine (0.156 g in 50 mL of EtOH) and Fe_2_Cl_3_ (0.5404 g in 2 mL of HCl 37% and 98 mL of water) were mixed in a concentration of 40:4:4 mL. A stock solution of 25 mM Trolox was prepared and successively diluted in a concentration between 200 and 10 µM. The solutions of the sample were prepared as explained for the DPPH· method. Next, 2700 µL of the FRAP solution and 300 µL of each concentration were mixed and allowed to react for 4 min. Then the absorbance was monitored at 593 nm. The results were related to the biomass intake during the extraction (in DM) and expressed as µmol Trolox per g biomass on dry matter base (µmol Trolox·g^−1^).

### 2.9. Calculations

The mass yield of the extraction processes was expressed as the ratio between the amount of extracted material and the biomass loaded in the extraction vessel on the dry matter base. In addition, the yield of carotenoids and phenolic compounds was determined. The carotenoids were treated individually. To ease the interpretation on the phenolic compounds, the different compounds were grouped in four classes: hydroxycinnamic acids (*p*-coumaric acids, caffeic, ferulic, chlorogenic), flavonoids (epicatechin, kaempferol, quercetin, quercitrin, aromadendrin, astragalin, avicularin, catechin, cynaroside, hyperin, isoquercitrin, naringenin, phlorizin, taxifolin), xanthonoids (mangiferin) and hydroxybenzoic acids (gallic and protocatechuic acids). All results were expressed as mass yield per g of biomass treated on the dry matter base.

### 2.10. Statistical Analysis

Most of the statistical analysis was performed by a design of experiments, using response surface methodology (RSM). At the start, a quadratic response model was considered for co-solvent concentration and temperature, whereas a linear response model was selected for the pressure, also including all interaction parameters. All values were subjected to analysis of variance (ANOVA) with a 95% confidence level. The final model (Equation (1)) was derived by removing all insignificant terms and used to determine the optimal conditions. For the final model, the values of the determination coefficient (R^2^) were also determined as an indication of the goodness of fit.

(1) $$Y=\beta_{o}+\overset{3}{\underset{i=1}{\sum }}\beta_{i}X_{i\;}+\overset{3}{\underset{i=1}{\sum }}\beta_{ii}X_{i}^{2}+\overset{3}{\underset{i=1}{\sum }}\overset{3}{\underset{j=i+1}{\sum }}\beta_{ij}X_{i}X_{j}$$

where Y is the dependent variable to be modelled (total carotenoids or individual carotenoids); $X_{i}$ and $X_{j}$ are the independent variables (temperature, pressure and co-solvent) and $\beta_{0}$ is the intercept; $\beta_{i}$ is the coefficient of linear effect; $\beta_{ii}$ and $\beta_{ij}$ are the coefficients of quadratic and interaction effects, respectively; and 3 is the number of variables. As explained, in this study, several models were developed for optimizing the extraction of total carotenoids as well as individual carotenoids. Additionally, surface plots from the fitted equations were generated in order to evaluate the effects of independent parameters and their mutual interaction.

To determine the significance of the differences between extraction conditions, the least significant difference (LSD) was calculated for each of the classes. The ANOVA was performed with at a 95% of confidence level. For the optimization study and the study on the different varieties, each extraction type was performed in duplicate, and the extracts were analyzed using the methodology as described above. In the figures, the average of both extractions is shown, whereby the difference calculated based on the percentage difference between the two replicate extractions is detailed in the legend. All statistical tests were conducted using STATISTICA XII, 2015 (Dell Inc., Tulsa, OK, USA).

## 3. Results and Discussion

In this research, the impact of pressure, temperature, type (acetone/ethanol) and concentration of the co-solvent was assessed on the extraction yield of bioactive compounds from mango by-products using SC-CO_2_ as the main solvent. In the first instance, the extraction procedure was optimized by maximizing the extraction of carotenoids through an RSM approach. Not only the total, but also the main individual carotenoids were assessed. The model was validated and, thereafter, the concentration of phenolics was evaluated for the selected conditions. In a second stage, the bioactive compounds of two mango varieties and two types of by-products, i.e., pulp and peel, were extracted at the optimized conditions and compared against a two-step reference extraction method using organic solvents.

### 3.1. Extraction as Function of Time

First, the extraction of the total carotenoids was analyzed as a function of time. The cumulative extraction yield was determined as a function of time for a temperature of 55 °C, a pressure of 35 MPa and using pure SC-CO_2_ as well as SC-CO_2_ modified with ethanol or acetone at different concentrations. In all cases, the extraction rate was found to decrease steadily with time (Supplementary Material SS.1). The declining concentration of extract in the outflowing solvent may be due to depletion in the extractable material or diffusional limitations due to mass transfer processes. After three hours, the steady state was reached, and the extraction was terminated in order to avoid degradation and isomerization due to temperature effects. This time was taken as a reference time for all further extractions.

### 3.2. Optimization of the Total Carotenoids Extraction via RSM Modelling

The influence of the co-solvent percentage, temperature and pressure on the overall carotenoid extraction yield was evaluated and optimized through a design of experiments, where temperature and co-solvent addition was studied at three levels and pressure at two levels. In order to have a representative concentration of carotenoids for the further experiments, a mixture of peel and pulp of the Kasturi variety was used. In total, 30 experiments were done, of which the conditions and their corresponding extracted total carotenoids content are given in Supplementary Material SS.2.

As explained above, the effects of three main variables on SC-CO_2_ extraction of carotenoids from mango were simultaneously evaluated using the RSM methodology to construct a model that allows for the selection of the best combination of conditions: temperature (*X*_1_), pressure (*X*_2_) and co-solvent percentage (*X*_3_). For each of the co-solvents, ethanol and acetone, a separate model was made. The carotenoid extraction (response) was mathematically modelled to predict the relationships between the working parameters and the extraction yield; however, all insignificant variables (95% level of significance) were removed. The regression models are shown below. As can be seen, the lack of fit for each case was found to be not significant. Moreover, the statistical analysis of the models is displayed in Supplementary Material SS.3:

$$\begin{array}{l} \text{Total carotenoids using EtOH as co-solvent}\;[µg/g\mathrm{DW}]\;=-25.115+0.881\;\times \;X_{1}+0.467\\ \;\times \;X_{2}-0.243\;\times \;X_{3}-0.008\;\times \;X_{1}^{2}+0.006\;\times \;X_{2}\;\times \;X_{3}+0.015\;\times \;X_{3}^{2}\;(R^{2}=0.97)\\ \text{Total carotenoids using acetone as co-solvent}\;[µg/g\mathrm{DW}]\;=-6.242+0.031\;\times \;X_{1}+0.670\\ \;\times \;X_{2}\;+0.079\;\times \;X_{3}-0.003\times X_{1}\;\times \;X_{2}-0.008\;\times \;X_{2}\;\times \;X_{3}\;(R^{2}=0.91) \end{array}$$

**Figure 1:**
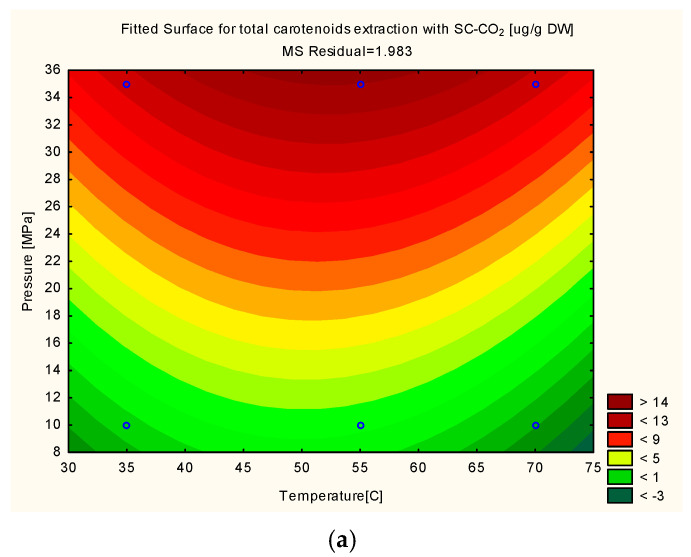

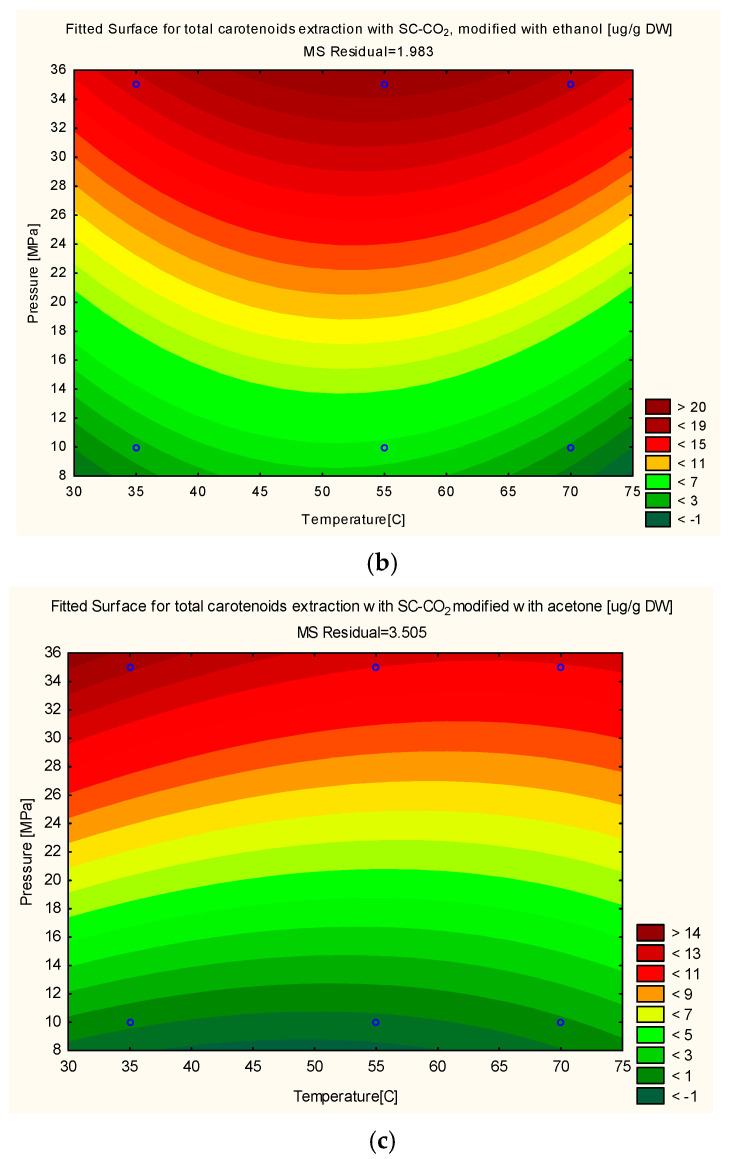
RSM for total carotenoids extracted with 0% co-solvent (**a**), 20% EtOH (**b**) and 20% acetone (**c**).

The three-dimensional surface plots provide a more comprehensive representation of the interaction between the working variables and the experimental response. Likewise, these figures facilitate the selection of the area within the graph where the optimal conditions are located. Hence, in Figure 1 it can be seen that when EtOH is used as co-solvent, low extractions are obtained at 35 °C, similarly as when the experiments were performed at a temperature of 70 °C. Similarly, high pressure allowed for an extraction of a higher yield, as compared to the yield obtained at 10 MPa. It should be mentioned that different behaviors were obtained when EtOH and acetone were separately used as co-solvents. Overall, more carotenoids were comparatively extracted with the use of EtOH as co-solvent than with the use of acetone.

**Figure 2:**
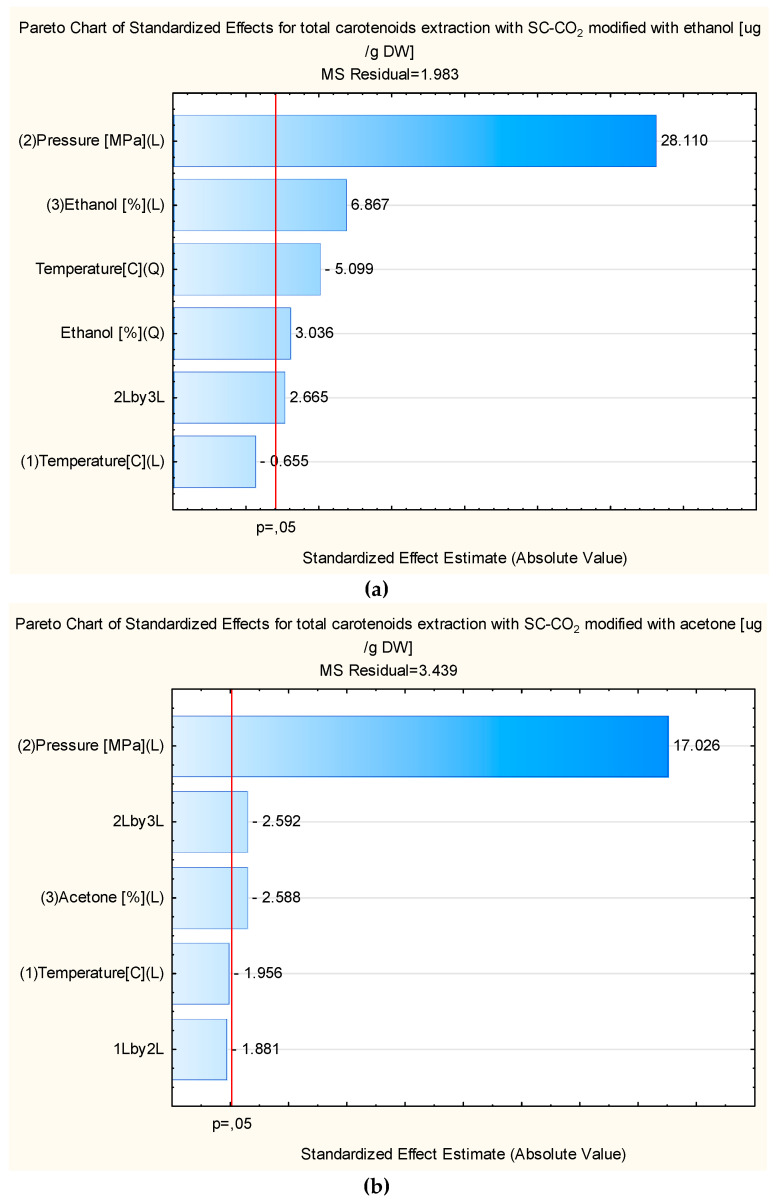
Pareto chart for total carotenoids extracted with ethanol (**a**) and acetone (**b**).

Figure 2 depicts the Pareto charts of the remaining linear, interaction and quadratic effects of the variables on the total yield. Overall, it can be observed that pressure and co-solvent addition are decisive in the concentration of total carotenoids recovered from mango.

In the extraction performed with ethanol as a co-solvent (Figure 2a), the linear effects of pressure (standardized effect of 28.1) and co-solvent addition (standardized effect of 6.9) were found to have a major and beneficial influence on the recovery of carotenoids. The temperature dependence was mostly dominated by the quadratic effect (−5.09), resulting in an optimum temperature around 50–55 °C. As shown in Figure 2a, other terms, such as the quadratic effect of concentration of ethanol and the interaction between pressure and concentration of ethanol, exerted a significant, though minor effect on the carotenoids’ extraction.

However, when the SC-CO_2_ was modified with acetone (Figure 2b), it was seen that only the linear effect of pressure (standardized effect of 17.0) positively influenced the recovery of carotenoids. Conversely, co-solvent concentration (standardized effect of −2.6), temperature (standardized effect −1.95) and the separate conjunctions of pressure and co-solvent and temperature and pressure (−2.59 and −1.88, respectively) exerted a significant but negative influence. Overall, these results show that pressure stands as an important factor for the extraction of compounds with SC-CO_2_ modified with acetone, where increasing temperature negatively influences on the overall yield.

The positive effect with pressure is well-known and associated with the higher density of the SC-CO_2_ [6]. The optimum temperature at intermediate values (55 °C), on the other hand, can be explained by the presence of two opposing mechanisms. It is well-known that mass transfer is improved with temperature because of the increase in the vapor pressure of the analytes present, facilitating their solvation [17]. The solubility, on the other hand, is diminished with temperature because of the decreasing density of the CO_2_ affecting the solvating power. The reduction observed in the yield above 55 °C is therefore likely caused by the fact that a decrease in solubility of the solvent has a greater effect compared to the increase in vapor pressure of the component.

The conditions to maximize the response were determined by the maxima of the generated plots (Figure 1a–c). By applying a temperature of 55 °C, pressure of 35 MPa and at 20% (*v*/*v*) of ethanol the highest extraction yield was obtained. With the application of such conditions, the predicted yield and the real yield were 19.52 µg·g^−1^ DW and 20.56 µg·g^−1^ DW, respectively. The ratio between the values obtained by the model and experimental value prediction was 94.9%.

### 3.3. Optimization of the Extraction Yield of the Individual Carotenoids via RSM Modelling

**Figure 3:**
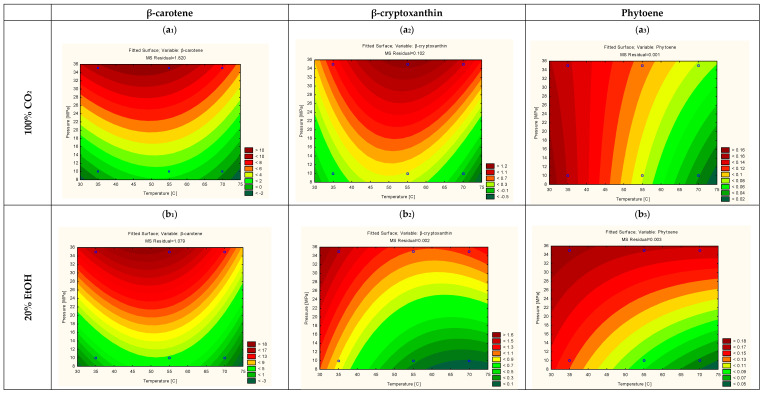

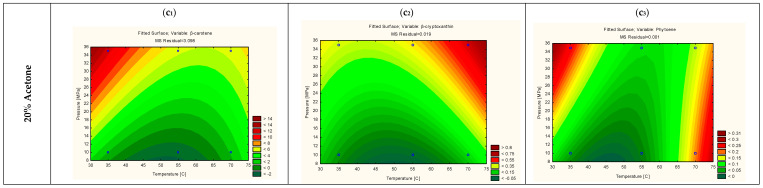
RSM for β-carotene extracted with 100% CO_2_ (**a1**), 20% EtOH (**b_1_**) and 20% Acetone (**c1**); β-cryptoxanthin extracted with 100% CO_2_ (**a2**), 20% EtOH (**b2**) and 20% Acetone (**c2**) and phytoene extracted with 100% CO_2_ (**a3**), 20% EtOH (**b3**) and 20% Acetone (**c3**).

To obtain a comprehensive knowledge for each of the carotenoids the influence of the extraction conditions was also assessed on each of the individual carotenoids in a similar way as described above for the total carotenoids (Table 1). In the extracts α and β-carotene, β-cryptoxanthin and phytoene were found to be the most abundant. Additionally, lower concentrations of cantaxanthin, capsanthin, lutein, violaxanthin and zeaxanthin were observed. For the most abundant carotenoids, an RSM model was constructed, whereby the insignificant parameters (confidence level 95%) were removed. The trends of the resulting models are shown in Figure 3. For all the compounds, pressure was found to be the most important factor, independent of the temperature, concentration or type of co-solvent, whose linear and/or quadratic effects had a lower but still significant influence on the extraction. The correlation (R^2^) was 0.81, 0.92 and 0.64 for β-cryptoxanthin, β-carotene and phytoene, respectively. As can be seen in Table 1, β-carotene is the most abundant carotenoid, and as expected, follows a similar behavior as reported above for the total carotenoids. Pressure and co-solvent are the major impacting parameters, whereby ethanol is more efficient than acetone (Figure 3(a1,b1,c1)). The presence of ethanol positively influences the amount of extracted β-carotene, leading to values up to 18.74 µg·g^−1^ DW under the optimal conditions. This increase can be explained based on molecular interactions between the solvent and the carotenoids via the Hansen solubility parameters (HSP). Tirado and Calvo (2019) [18], calculated that the HSP of β-carotene and SC-CO_2_ modified with ethanol are very similar, explaining the suitability of the latter as an extracting medium. Specifically, the distance (Ra) between the HSP of the mixture CO_2_ + EtOH and those of β-carotene when extracted at 40 and 50 °C were smaller when compared with acetone and other organic solvents, specifically 12.14 and 13.57 for each temperature, respectively. It must be noted that this model only considers the affinity between the solute and solvent and does not consider the competitive interactions that exist in the matrix.

As for the other carotenoids, β-cryptoxanthin had a very similar behavior as β-carotene, as can be seen in Figure 3(a2,b2). Phytoene, on the other hand, had a different behavior, since their highest extraction yield was obtained at 35 °C. This different behavior is likely due to a higher sensitivity to isomerization or oxidative degradation with increasing temperature [19]. At a temperature of 55 °C and with EtOH as co-solvent, both β-carotene and β-cryptoxanthin have their highest yield of 18.74 and 2.23 µg·g^−1^ DW, respectively. These values are very close to the predicted 17.45 µg·g^−1^ DW and 1.55 µg·g^−1^ DW, respectively. Nevertheless, if extracts rich in phytoene are targeted, a lower operating temperature of 35 °C is better suited, resulting in a yield of 0.26 µg·g^−1^ DW, which is comparable to the predicted value of 0.23 µg·g^−1^ DW in the model.

### 3.4. Validation of the Model for Carotenoids Extraction and Behavior of the Phenolic Compounds

As the phenolic compounds are also valuable constituents that may be co-extracted, their presence was also evaluated in the extracts obtained at this subset of conditions. To validate the model obtained, the extractions at the optimum as well as at a subset of conditions with higher carotenoid yield were repeated. As a pressure of 35 MPa resulted in good carotenoid extraction, the subset was focused on variation of temperature and co-solvent, while keeping the pressure mostly constant.

**Figure 4:**
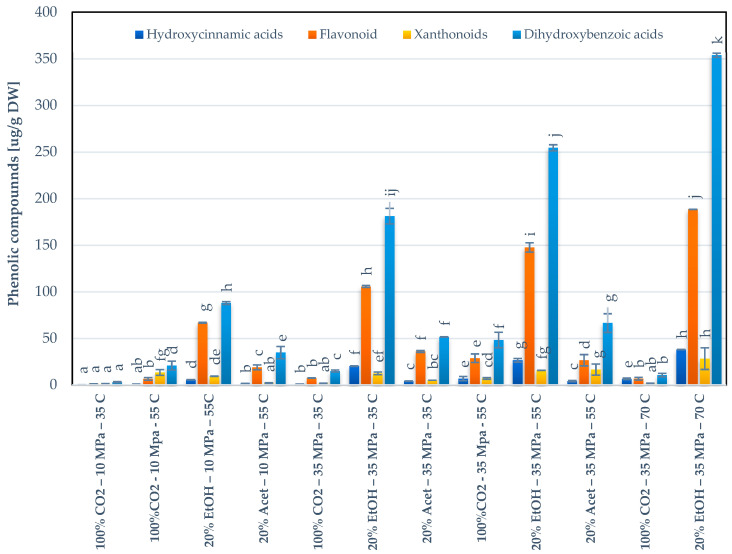
Total phenolic compounds (evaluated as sum of individual compounds) obtained in the confirmation data set. Values are mean ± relative difference (*n* = 2). Means with different letters indicate statistically significant differences between extraction conditions and the concentration of phenolics of the same class (*p <* 0.05).

The results obtained for phenolics are described in Figure 4. The modification of SC-CO_2_ with 20% ethanol enhanced the extraction yield of phenolics when compared with pure CO_2_ and acetone. On the other hand, the temperature and the extraction yield of phenolics were directly proportional. The higher yield was obtained at 70 °C (608.13 µg·g^−1^ DW). The values presented in this study are higher than those provided by Garcia-Mendoza et al. (2015) [8]. Moreover, Meneses et al. (2015) [20], indicated that environmental factors like UV radiation, factors inherent to the process like storage and the cultivar itself affect the phenolic content of mango. The higher yield obtained with SC-CO_2_ modified with organic solvents can likely be attributed to the predominance of the vapor pressure effect over the density effect. The solvent’s viscosity and the surface tension decrease at higher temperatures; thus, its penetration into the matrix is enhanced. Moreover, the molecular interactions (e.g., solute-matrix, in the form of sorption isotherm/isobar models) are broken; therefore, the desorption energy of the target compounds is reduced [21]. Moreover, it is possible that the particle cell walls are damaged, which can result in an increment in the mass transfer [22]. It should also be indicated that certain phenolic compounds like gallic and vanillic acids are relatively stable, while catechin is the most unstable when exposed to temperatures from 80 to 100 °C, so that degradation effects are expected to be less important as in the case of carotenoids [23].

As stated, the addition of co-solvents significantly improved the extraction yield of phenolics in comparison to the extraction performed with pure CO_2_. In this line, higher yields were obtained with EtOH than with acetone. This enhancement could be likely explained by the higher hydrogen donor strength of ethanol than acetone (0.83 vs. 0.08, respectively) [24], which is related to the extraction yield. Besides the processing parameters, Molino et al. (2020) [6] explained there are inherent factors that affect the solubility of the compounds in SC-CO_2_, like the molecular mass and structure of the compounds, since the complexity of the molecule produces a reduction in the solubility.

For a better understanding of the behavior of different phenolic classes, the individual compounds were separated in four groups: dihydroxybenzoic acids, flavonoids, hydroxycinnamic acids and xanthonoids (see Figure 4). Dihydroxybenzoic acids were quantified between 2.90 and 353.81 µg·g^−1^ DW. Flavonoids had a total yield ranging from 1.07 to 188.39 µg·g^−1^ DW. Within this group, quercetin was the most concentrated one, representing between 13 and 55 wt. % of the total class. With respect to hydroxycinnamic acids, ferulic acid represented between 28 and 86 wt. % of the total class. The overall yield of hydroxycinnamic acids was found to be between 0.15 µg·g^−1^ DW and 37.78 µg·g^−1^ DW. Finally, mangiferin was the only representative of the xanthones group, whose yield was between 1.1 and 28.15 µg·g^−1^ DW. The profile and the principal compounds found in this study were similar to those reported for mango by Schieber et al. (2000) and Agatonovic-Kustrin et al. (2018) [25,26] (chromatograms of the analysis of phenolics and carotenoids are displayed in Supplementary Material SS.4).

### 3.5. Extraction of Carotenoids and Phenolic Compounds at Optimized Conditions on By-Products Obtained from Different Ecuadorian Mango Varieties and Assessment of the Antioxidant Activities

After optimizing the extraction conditions, the concentration of carotenoids and phenolics was compared in extracts performed with SC-CO_2_ and organic solvents in the peel and pulp of two different mango varieties: “Kent” and “Haden”. Moreover, the DPPH· and FRAP methods were applied to assess the antioxidant activity of the extracts.

#### 3.5.1. Carotenoids

Given that carotenoids are secondary metabolites, their presence and concentration can be different depending on the variety and the environmental conditions of the vegetal matrix. Therefore, the effect of extraction conditions (SC-CO_2_ or organic solvent extraction) was evaluated in the variability of the carotenoids profile in the by-products of two different mango varieties. In this study, it was observed that the concentration of the carotenoids was influenced by the varieties of mango and the type of by-products analyzed. Overall, the concentration of total carotenoids on peels of the different varieties were between 44.71 and 65.66 µg·g^−1^ DW on the samples extracted with organic solvents, while for the extracts performed with SC-CO_2_, the concentration was between 43.12 and 53.87 µg·g^−1^ DW. Lower concentrations were registered in the pulp, with 17.35 to 32.23 µg·g^−1^ DW and 13.09 to 21.99 µg·g^−1^ DW in the samples extracted with organic solvents and SC-CO_2_, respectively.

The varietal differences in the concentration of carotenoids in mango have been previously reported. Mercadante and Rodriguez-Amaya (1998) [27], found a concentration of β-carotene of 5.8 µg·g^−1^ DW in the “Tommy” pulp variety, and of 15 µg·g^−1^ DW in the “Keitt” pulp variety, which is significantly lower than the concentration reported in the present study for the by-products of all varieties analyzed. Similarly, Pott et al. (2003) [28] reported variations of the carotenoids present in the flesh from three cultivars of mangoes. The concentration of β-carotene in the “Kent” and “Tommy” variety was 46 and 37 µg·g^−1^ DW, respectively, similar to those obtained in the varieties utilized in this study. These variations could be due to both genotypic [29] and environmental factors [30].

It was also found that the carotenoid concentration in the peels was between 1.3- and 4.1-fold higher than in the pulp. This could be due to the active role of carotenoids in photosystem assembly where they act as a photoprotector. The higher exposure to sunlight induces an increase in carotenogenesis, leading to higher concentrations of carotenoids in the peels [30], making the peels a valuable resource for bioactive compound recovery.

As can be seen in Table 2, the use of organic solvents allowed for a higher extraction yield of total carotenoids (between 6% and 46%). The concentration of total carotenoids determined for the extracts performed with SC-CO_2_ in all the varieties in this study was similar to the range reported by Haque et al. (2015) [31] in different mango varieties from Bangladesh extracted with *n*-hexane, but lower than the results reported by Garcia-Mendoza et al. (2015) [8] in mango peels with non-modified SC-CO_2_. The concentration of β-carotene, β-cryptoxanthin and lutein (Table 2) was lower than the concentration reported in mango “Ataulfo” [32]. In addition, the solid remnants after extraction with SC-CO_2_ could be used for several applications. In another study, de Andrade Lima et al. (2018) [33] found that carbohydrates, proteins and lipids are retained within the biomass after SFE. Consequently, the extracted biomass could potentially be used as source of such macronutrients for different uses (e.g., stabilizers or emulsifiers), and in a subsequent process for transformation into platform or biofuels.

#### 3.5.2. Phenolic Compounds

The health-promoting activities of phenolic compounds are of interest for researchers and industry. Similar to carotenoids, phenolics are secondary metabolites whose profile can vary depending on the extraction technique. Hence, SC-CO_2_ and solvent extraction were compared in terms of concentration of individual phenolics in extracts obtained from mango by-products, with the first priority focused on carotenoids.

**Figure 5:**
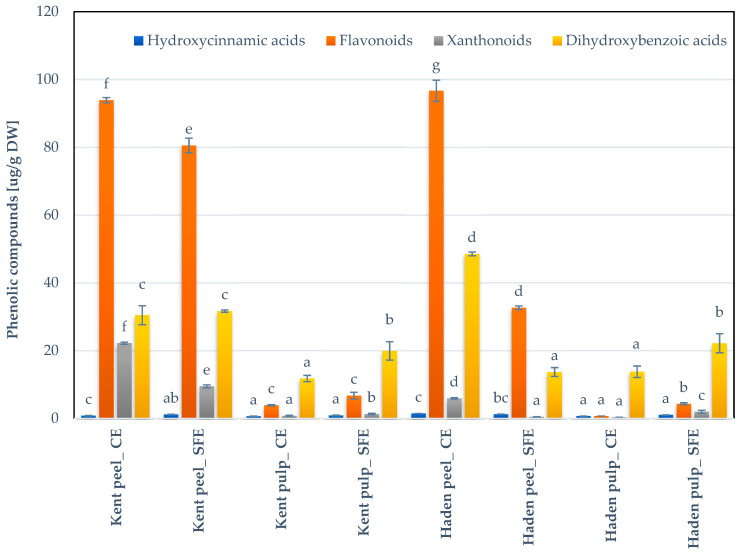
Total phenolic compounds (evaluated as sum of individual compounds) obtained in the different mango varieties. Values are mean ± relative difference (*n* = 2). Means with different letters indicate statistically significant differences between extraction method and the concentration of phenolics in the same fraction and variety of mango (*p <* 0.05). CE = conventional extraction. SFE = supercritical fluid extraction.

In total, 21 different phenolic compounds were found in the mango matrix, whereby the flavonoids and phenolic acids were the most important ones (see Figure 5). Nevertheless, the profile of the individual compounds varied significantly, not only among the different extraction techniques, but also among the fraction assessed (Table 3).

Overall, the two-step organic solvent extraction resulted in a higher yield of phenolics. The total yield of phenolics, determined as the sum of the individual compounds, ranged between approximately 147.28 and 152.52 µg·g^−1^ DW in the peel extracts, and between 15.42 µg·g^−1^ DW and 17.06 µg·g^−1^ DW in the pulp extracts. In general, gallic acid was the most abundant compound, in line with other studies [34]. The extraction of the peels resulted in a yield of 29.70 µg·g^−1^ DW for the variety “Kent” and 48.21 µg·g^−1^ DW (for the variety “Haden”), while the yields of the corresponding pulps were 11.55 and 13.21 µg·g^−1^ DW for the “Kent” and “Haden” varieties, respectively. The results obtained in the peels are similar to those reported by Yao et al. (2020) [35], who found between 84.86 and 147.99 µg·g^−1^ DW of mango pulp (variety “Sensation”). The hydroxybenzoic acids were quantified up to 48.5 µg·g^−1^ DW in the peels of the variety “Haden”, and the pulp was characterized by a yield of 13.68 µg·g^−1^ DW. The flavonoids were the second group of phenolic compounds present in the mango by-products. The extraction of the peels of the “Haden” variety resulted in a yield of 96.65 µg·g^−1^ DW, as compared to 93.89 µg·g^−1^ DW for the “Kent” variety. In the pulp, up to 3.89 µg·g^−1^ DW was quantified, which is lower than reported by Rumainum et al. (2018) [36] in the pulp of different mango cultivars from Thailand (up to 670 µg·g^−1^ DW). Finally, the yield of mangiferin (5.90 to 22.19 µg·g^−1^ DW and 0.33 to 0.78 µg·g^−1^ DW in the peels and pulps, respectively), a xanthone glycoside with possible applications in auto-immune diseases (e.g., psoriasis, rheumatoid arthritis and dermatitis) [37], was lower than the results presented by Vithana et al. (2019) [34] in the pulp (13 µg·g^−1^ DW) and in the peel (194 µg·g^−1^) of the mango “Kensington Pride” in the extracts obtained with 80% methanol. Overall, it could be observed that the variety “Kent” exceled in the concentration of mangiferin as compared with the other varieties.

As expected, the extraction technique affected the phenolic profile of the mango fractions, in which higher contents of phenolic compounds were determined in extracts performed with organic solvents than the content in extracts performed with SC-CO_2_. Hence, SC-CO_2_ extraction led to a significantly lower yield of phenolics. The total compounds were between 48.01 and 122.76 µg·g^−1^ DW in the peel extracts, while for the pulp this yield was between 28.80 µg·g^−1^ DW and 29.45 µg·g^−1^ DW. In general, the quantification of phenolics in peels was between 16% and 68% lower when SC-CO_2_ was used as solvent, while in pulp the extraction with SC-CO_2_ it was significantly higher (40% to 47%). The trends in the different extracts remained similar to the ones obtained in the extracts obtained with organic solvents. Moreover, no specificity for the compounds was found towards the extraction method used.

According to de O. Silva et al. (2019) [38], the differences evidenced in the yield between the two extraction methods were mainly due to the polarity of the extraction media, meaning that higher amounts of phenolics were dragged out to the solvent. Moreover, the high phenolic contents obtained with organic solvents may be due to the better interaction, in the form of hydrogen bonds, on the polar sites of the phenolics with the solvent. Additionally, it has been shown that interferences could be avoided by additional sample purification procedures for the exclusion of non-target substances (e.g., terpenes, vitamins, pigments and fats) [39]. In general, data on the phenolic composition of mango extracts obtained by SC-CO_2_ are limited.

#### 3.5.3. Antioxidant Activity

As can be seen in Table 4, the antioxidant activity of the extracts, as determined by the DPPH· and FRAP essays, is influenced by both the type of extraction as well as the by-product considered. The two assays show an opposite response, which relates to their methodology. With increasing antioxidant activity, in DPPH·, more radicals are neutralized, resulting in a lower absorbance. Conversely, in FRAP, more Fe^3+^ is reduced to the colored Fe^2+^ complex, giving a higher absorbance.

Both methods indicate a higher antioxidant activity in peel extracts, with a value between 0.63 and 5.68 µg·mL^−1^ for DPPH, and 2.46 and 17.45 µmol Trolox·g^−1^ for FRAP, depending on the extraction methodology. The pulp extracts possessed systematically lower antioxidant activities with values ranging between 3.68 and 17.36 µg·mL^−1^ for DPPH, and 0.91 µmol and 2.60 µmol Trolox·g^−1^ for FRAP. The antioxidant activity was systematically higher for “Haden” as compared to the “Kent” extracts, which can be attributed to their higher concentration in phenolics and carotenoids (Table 2, Figure 5).

Studies have demonstrated that the antioxidant activity of vegetal materials is due to the interaction of carotenoids and polyphenols [40]. As discussed above, catechin, gallic acid and isoquercitrin were found to be the main polyphenols in the two mango varieties. Regression analysis between their concentration and the observed antioxidant activity showed a correlation of 0.88, 0.82 and 0.71, respectively, suggesting their importance to the observed antioxidant activity. This is in agreement with other studies where the antioxidant activity of mango was primarily attributed to the presence of gallic acid and flavonoids [41,42]. Through their chelating properties, these compounds likely reduce the availability of metal ions by the formation of metal complexes that otherwise would be involved in the formation of radicals [43]. As for carotenoids, mostly β-carotene in the mango extracts, their scavenging activity is attributed to the double bonds on the molecular structure that are expected to act as effective quenchers [44]. 

SFE extracts have in general lower antioxidant activity as compared to the extracts obtained via the more complex solvent extraction system. The latter is based on two successive steps involving solvents with a range of different polarities, possibly reducing biomass interactions, (e.g., the weak non-covalent interaction between plant proteins and phenolic compounds) [45], leading to a more complete extraction. Moreover, other compounds with antioxidant activity could have been co-extracted. However, the large solvent use and associated complex solvent recovery makes the conventional extraction, as it was performed in this research, not suitable for upscaling. Conversely, the more environmentally friendly SFE based on a single step with integrated SC-CO_2_ recovery has been shown to be scalable for the extraction of plant-origin bioactive compounds and is considered to be a promising alternative.

## 4. Conclusions

Through the results delivered in the present study, it was possible to evaluate the effect of process parameters on the extraction of carotenoids and phenolics from mango by-products via supercritical CO_2_ extraction. The modeling of the extraction behavior for the main carotenoids was presented as part of the overall assessment for a complete biorefinery process of the by-products. As expected, β-carotene was the main carotenoid in the mango matrix, while the phenolic composition was diverse depending on the conditions applied for the extraction. The modification of CO_2_ with 20% ethanol and with mild and high temperatures resulted in a higher extraction yield of carotenoids and phenolics, respectively. Nevertheless, the extraction yield and antioxidant activity were lower when compared with conventional extraction techniques with organic solvents. Overall, the outcomes of this study show the potential of SC-CO_2_ modified with ethanol for the recovery of bioactive compounds from mango by-products as a first step of a complete green biorefinery process.

## Figures and Tables

**Table 1:** Quantification of the amount of carotenoids extracted with SC-CO_2_ in the optimization set of experiments per amount of biomass extracted from the mango variety Kasturi.

	10 MPa				35 MPa			
	α-Carotene [µg·g^−1^ DW]	β-Cryptoxantin [µg·g^−1^ DW]	β-Carotene [µg·g^−1^ DW]	Phytoene [µg·g^−1^ DW]	α-Carotene [µg·g^−1^ DW]	β-Cryptoxantin [µg·g^−1^ DW]	β-Carotene [µg·g^−1^ DW]	Phytoene [µg·g^−1^ DW]
35 °C; 100% CO_2_	<LOQ	0.24 ± 0.01 ^d^	0.31 ± 0.02 ^ab^	0.16 ± 0.04 ^ef^	1.03 ± 0.07 ^ef^	0.65 ± 0.01 ^abc^	8.96 ± 0.23 ^d^	0.13 ± 0.00 ^abc^
35 °C; 10% EtOH	<LOQ	0.14 ± 0.07 ^cd^	0.17 ± 0.02 ^a^	<LOD	1.24 ± 0.11 ^g^	1.52 ± 0.27 ^ef^	6.81 ± 0.19 ^c^	0.19 ± 0.03 ^cde^
35 °C; 20% EtOH	<LOQ	0.91 ± 0.08 ^e^	1.52 ± 0.08 ^c^	0.11 ± 0.02 ^d^	1.51± 0.09 ^h^	1.49 ± 0.17 ^ef^	15.28 ± 0.92 ^h^	0.19 ± 0.01 ^de^
35 °C; 10% Ac	<LOQ	0.03 ± 0.00 ^a^	0.25 ± 0.08 ^ab^	0.08 ± 0.02 ^cd^	0.26 ± 0.03 ^b^	0.33 ± 0.07 ^a^	11.36 ± 0.37 ^e^	0.26 ± 0.03 ^e^
35 °C; 20% Ac	<LOQ	0.02 ± 0.00 ^a^	0.13 ± 0.00 ^a^	0.04 ± 0.02 ^bc^	0.39 ± 0.01 ^c^	0.39 ± 0.01 ^ab^	12.16 ± 0.38 ^f^	0.23 ± 0.01 ^de^
55 °C; 100% CO_2_	<LOQ	0.04 ± 0.01 ^a^	0.11 ± 0.02 ^a^	0.05 ± 0.01 ^bc^	1.32 ± 0.02 ^g^	1.68 ± 0.03 ^f^	11.63 ± 1.14 ^ef^	0.14 ± 0.04 ^bcd^
55 °C; 10% EtOH	<LOQ	0.07 ± 0.00 ^ab^	1.44 ± 0.17 ^c^	0.06 ± 0.02 ^bc^	1.01 ± 0.12 ^e^	2.23 ± 0.14 ^g^	13.41 ± 0.42 ^g^	0.07 ± 0.00 ^ab^
55 °C; 20% EtOH	<LOQ	0.19 ± 0.01 ^de^	3.70 ± 0.87 ^d^	0.15 ± 0.02 ^e^	1.13 ± 0.14 ^f^	1.13 ± 0.04 ^de^	18.74 ± 0.21 ^i^	0.10 ± 0.07 ^ab^
55 °C; 10% Ac	<LOQ	0.10 ± 0.02 ^bc^	0.11 ± 0.02 ^a^	0.09 ± 0.02 ^d^	0.39 ± 0.08 ^c^	1.52 ± 0.04 ^ef^	5.81 ± 0.23 ^b^	0.09 ± 0.02 ^ab^
55 °C; 20% Ac	<LOQ	0.13 ± 0.00 ^c^	0.22 ± 0.00 ^ab^	0.03 ± 0.00 ^ab^	0.27 ± 0.02 ^b^	0.24 ± 0.18 ^a^	3.35 ± 0.34 ^a^	0.07 ± 0.06 ^ab^
70 °C; 100% CO_2_	<LOQ	0.07 ± 0.04 ^ab^	<LOD	0.05 ± 0.04 ^bc^	0.41 ± 0.04 ^cd^	0.81 ± 0.09 ^cd^	6.23 ± 0.52 ^bc^	0.06 ± 0.02 ^a^
70 °C; 10% EtOH	<LOQ	0.07 ± 0.01 ^ab^	0.01 ± 0.00 ^a^	0.05 ± 0.00 ^bc^	0.52 ± 0.00 ^d^	1.29 ± 0.10 ^ef^	5.80 ± 0.57 ^b^	0.08 ± 0.01 ^ab^
70 °C; 20% EtOH	<LOQ	0.11 ± 0.00 ^bc^	0.61 ± 0.05 ^b^	0.02 ± 0.00 ^ab^	0.38 ± 0.05 ^c^	1.13 ± 0.00 ^de^	11.08 ± 0.20 ^e^	0.19 ± 0.04 ^cde^
70 °C; 10% Ac	<LOQ	0.11 ± 0.01 ^bc^	0.02 ± 0.01 ^a^	0.04 ± 0.00 ^b^	0.05 ± 0.00 ^a^	0.83 ± 0.49 ^cd^	8.65 ± 0.03 ^d^	0.07 ± 0.00 ^ab^
70 °C; 20% Ac	<LOQ	0.07 ± 0.01 ^ab^	0.03 ± 0.00 ^a^	0.19 ± 0.02 ^g^	0.04 ± 0.00 ^a^	0.74 ± 0.07 ^bcd^	6.46 ± 0.25 ^bc^	0.13 ± 0.08 ^bcd^

Values are mean ± relative difference (*n* = 2). LOD = limit of quantification. Means with different letters indicate statistically significant differences between extraction conditions and the specific compound in the mango matrix (*p <* 0.05).

**Table 2:** Amount of the various carotenoids extracted with CE and SFE from the pulp and peel of different mango varieties.

		Total Carotenoids [µg·g^−1^ DW]	α-Carotene [µg·g^−1^ DW]	β-Kryptoxanthin [µg·g^−1^ DW]	β-Carotene [µg·g^−1^ DW]	Lutein [µg·g^−1^ DW]	Violaxanthin [µg·g^−1^ DW]	Phytoene [µg·g^−1^ DW]	Phytofluene [µg·g^−1^ DW]	Zeaxanthin [µg·g^−1^ DW]
Kent peel	CE *	44.71 ± 0.02 ^a^	0.99 ± 0.01 ^a^	0.28 ± 0.06 ^a^	34.38 ± 1.89 ^a^	1.26 ± 0.03 ^a^	0.08 ± 0.01 ^b^	0.43 ± 0.02 ^a^	0.76 ± 0.01 ^b^	0.26 ± 0.06 ^a^
	SFE **	43.12 ± 6.53 ^b^	1.45 ± 0.59 ^b^	0.26 ± 0.01 ^a^	35.52 ± 4.09 ^a^	1.69 ± 0.12 ^b^	0.17 ± 0.02 ^c^	0.57 ± 0.04 ^b^	0.92 ± 0.02 ^c^	0.33 ± 0.02 ^b^
Kent pulp	CE	32.23 ± 0.99 ^a^	1.36 ± 0.34 ^a^	0.76 ± 0.06 ^a^	24.02 ± 0.49 ^a^	0.94 ± 0.14 ^a^	<LOQ	0.01 ± 0.02 ^a^	<LOQ	0.24 ± 0.03 ^a^
	SFE	22.00 ± 0.12 ^b^	0.38 ± 0.19 ^b^	0.33 ± 0.01 ^b^	17.87 ± 1.88 ^b^	<LOQ	<LOQ	0.14 ± 0.02 ^b^	<LOQ	<LOQ
Haden peel	CE	65.66 ± 4.45 ^a^	7.09 ± 0.59 ^a^	1.50 ± 0.21 ^a^	46.47 ± 1.13 ^a^	1.29 ± 0.00 ^a^	0.45 ± 0.13 ^b^	0.69 ± 0.14 ^b^	0.78 ± 0.01 ^b^	0.14 ± 0.01 ^a^
	SFE	53.87 ± 0.87 ^b^	2.52 ± 0.04 ^b^	1.12 ± 0.09 ^b^	33.30 ± 4.16 ^b^	1.05 ± 0.07 ^b^	0.14 ± 0.03 ^b^	0.20 ± 0.06 ^a^	0.19 ± 0.00 ^c^	0.12 ± 0.02 ^b^
Haden pulp	CE	17.35 ± 0.33 ^a^	0.16 ± 0.05 ^a^	0.24 ± 0.03 ^a^	11.33 ± 8.45 ^a^	0.07 ± 0.00 ^a^	0.03 ± 0.00 ^b^	0.28 ± 0.01 ^a^	0.23 ± 0.01 ^b^	0.03 ± 0.01 ^a^
	SFE	13.09 ± 0.91 ^b^	0.17 ± 0.06 ^a^	0.26 ± 0.08 ^a^	8.72 ± 0.22 ^a^	0.04 ± 0.00 ^a^	0.01 ± 0.00 ^c^	0.19 ± 0.02 ^a^	0.24 ± 0.00 ^b^	0.01 ± 0.01 ^b^

* CE = conventional extraction. ** SFE = supercritical fluid extraction (20% EtOH, 55 °C, 35 MPa). Values are mean ± relative difference (*n* = 2). LOQ = limit of quantification. Means with different letters in the same row indicate statistically significant differences between the concentration of the compounds as function of the extraction method (*p <* 0.05).

**Table 3:** Main phenolics identified in the peel and pulp of the mango varieties Kent and Haden.

		Kent Peel		Kent Pulp		Haden Peel		Haden Pulp	
		CE *	SFE **	CE	SFE	CE	SFE	CE	SFE
Hydroxycinnamic acids	Caffeic acid	0.19 ± 1.03 ^b^	0.27 ± 4.82 ^c^	0.16 ± 6.90 ^a^	0.22 ± 13.26 ^b^	0.39 ± 0.22 ^e^	0.24 ± 0.05 ^b^	0.30 ± 1.46 ^d^	0.41 ± 3.53 ^e^
	Chlorogenic acid	<LOQ	<LOQ	<LOQ	<LOQ	0.17 ± 1.36 ^b^	0.02 ± 8.97 ^a^	<LOQ	<LOQ
	Ferulic acid	0.14 ± 16.09 ^c^	0.18 ± 3.77 ^d^	0.05 ± 0.33 ^a^	0.08 ± 9.50 ^b^	0.25 ± 0.95 ^f^	0.21 ± 1.73 ^e^	0.15 ± 5.99 ^c^	0.27 ± 8.88 ^g^
	*p*-Coumaric acid	0.46 ± 8.79 ^c^	0.71 ± 3.63 ^f^	0.43 ± 6.79 ^c^	0.57 ± 9.39 ^d^	0.66 ± 1.59 ^e^	0.76 ± 2.65 ^g^	0.22 ± 5.08 ^a^	0.35 ± 1.87 ^b^
Flavonoids	Cymaroside	0.06 ± 4.63 ^b^	0.02 ± 4.00 ^a^	<LOQ	<LOQ	0.10 ± 3.04 ^c^	<LOQ	<LOQ	<LOQ
	Aromadendrin	0.02 ± 4.04 ^e^	0.03 ± 4.08 ^f^	0.01 ± 13.20 ^d^	0.01 ± 10.95 ^c^	0.01 ± 0.67 ^a^	0.02 ± 1.82 ^b^	<LOQ	0.01 ± 6.28 ^a^
	Avicularin	10.53 ± 3.18 ^c^	10.41 ± 1.26 ^c^	0.02 ± 65.27 ^a^	0.27 ± 18.11 ^a^	10.97 ± 4.16 ^d^	4.49 ± 4.05 ^b^	0.01 ± 1.51 ^a^	0.26 ± 40.31 ^a^
	Astragalin	0.86 ± 2.50 ^d^	1.94 ± 4.04 ^e^	<LOQ	0.11 ± 18.29 ^a^	0.56 ± 4.29 ^b^	0.64 ± 3.96 ^c^	<LOQ	0.11 ± 24.90 ^a^
	Hyperin	26.57 ± 4.89 ^e^	23.97 ± 4.19 ^d^	<LOQ	1.40 ± 17.65 ^b^	27.98 ± 4.31 ^e^	10.73 ± 2.05 ^c^	0.07 ± 3.22 ^a^	1.58 ± 2.31 ^b^
	Phlorizin	0.18 ± 3.88 ^d^	0.16 ± 2.82 ^c^	<LOQ	0.01 ± 12.79 ^a^	0.18 ± 0.05 ^d^	0.06 ± 3.75 ^b^	<LOQ	<LOQ
	Taxifolin	0.37 ± 1.64 ^e^	0.77 ± 2.68 ^f^	0.03 ± 7.59 ^a^	0.06 ± 16.93 ^b^	0.15 ± 1.33 ^c^	0.27 ± 0.75 ^d^	0.02 ± 3.66 ^a^	0.03 ± 5.55 ^a^
	Naringenin	0.15 ± 12.59 ^e^	0.15 ± 4.21 ^e^	0.02 ± 7.15 ^a^	0.03 ± 14.16 ^c^	0.16 ± 2.79 ^d^	0.14 ± 3.79 ^b^	<LOQ	0.01 ± 7.85 ^a^
	Epicathecin	0.14 ± 1.49 ^d^	0.06 ± 4.69 ^c^	0.03 ± 4.99 ^b^	0.03 ± 11.95 ^b^	0.14 ± 3.86 ^d^	0.01 ± 1.16 ^a^	0.01 ± 15.69 ^a^	<LOQ
	Kaempferol	0.04 ± 78.95 ^a^	0.07 ± 18.32 ^b^	<LOQ	<LOQ	0.07 ± 1.24 ^b^	0.09 ± 1.56 ^c^	<LOQ	0.07 ± 28.42 ^b^
	Isoquercitrin	27.66 ± 1.50 ^g^	26.81 ± 1.95 ^f^	0.11 ± 31.36 ^b^	1.11 ± 17.34 ^c^	27.47 ± 2.01 ^f^	11.11 ± 0.60 ^e^	0.08 ± 6.35 ^a^	1.26 ± 2.51 ^d^
	Quercetin	0.99 ± 13.54 ^c^	1.67 ± 5.37 ^d^	<LOQ	0.29 ± 38.82 ^a^	2.78 ± 0.71 ^f^	1.92 ± 5.28 ^e^	<LOQ	0.53 ± 9.01 ^b^
	Quercitrin	5.03 ± 5.75 ^e^	4.34 ± 3.12 ^d^	0.01 ± 26.32 ^a^	0.08 ± 21.53 ^b^	5.01 ± 1.27 ^e^	1.70 ± 0.68 ^c^	<LOQ	0.08 ± 25.13 ^b^
	Catechin	21.28 ± 0.99 ^e^	10.13 ± 1.43 ^d^	3.67 ± 3.75 ^c^	3.31 ± 8.64 ^c^	21.06 ± 3.95 ^e^	1.45 ± 4.50 ^b^	<LOQ	0.35 ± 1.20 ^a^
Xanthonoids	Mangiferin	22.19 ± 1.44 ^h^	9.47 ± 4.54 ^g^	0.78 ± 16.39 ^c^	1.30 ± 15.86 ^d^	5.90 ± 3.45 ^f^	0.48 ± 10.95 ^b^	0.34 ± 0.87 ^a^	1.96 ± 23.30 ^e^
Dihydroxybenzoic acids	Gallic acid	29.70 ± 8.11 ^e^	30.45 ± 0.99 ^e^	11.55 ± 7.74 ^a^	18.98 ± 13.52 ^c^	48.21 ± 1.15 ^f^	13.13 ± 7.87 ^b^	13.21 ± 8.33 ^b^	21.15 ± 11.89 ^d^
	Protocatechuic acid	0.72 ± 70.58 ^d^	1.18 ± 2.53 ^f^	0.22 ± 14.14 ^a^	0.94 ± 16.33 ^e^	0.29 ± 5.50 ^b^	0.55 ± 60.96 ^c^	0.56 ± 18.65 ^c^	1.01 ± 27.49 ^f^

* CE = conventional extraction. ** SFE = supercritical fluid extraction (20% EtOH, 55 °C, 35 MPa). Values are mean ± relative difference (*n* = 2). LOQ = limit of quantification. Means with different letters in the same row indicate statistically significant differences in the concentration of the compounds as function of the extraction method (*p* < 0.05).

**Table 4:** Antioxidant activity determined by the DPPH· and FRAP methods.

		DPPH [µg·mL^−1^]	FRAP [µmol Trolox g^−1^]
Kent peel	CE *	0.63 ± 0.00 ^a^	10.89 ± 0.27 ^a^
	SFE **	5.68 ± 0.08 ^b^	2.46 ± 0.15 ^b^
Kent pulp	CE	7.97 ± 0.24 ^a^	2.60 ± 0.15 ^a^
	SFE	3.68 ± 0.08 ^b^	2.06 ± 0.02 ^b^
Haden peel	CE	0.64 ± 0.02 ^a^	17.45 ± 0.13 ^a^
	SFE	1.34 ± 0.03 ^b^	4.25 ± 0.16 ^b^
Haden pulp	CE	6.11 ± 0.27 ^a^	2.09 ± 0.03 ^a^
	SFE	17.36 ± 0.63 ^b^	0.91 ± 0.02 ^b^

* CE = conventional extraction. ** SFE = supercritical fluid extraction (20% EtOH, 55 °C, 35 MPa). Values are mean ± relative difference (*n* = 2). Means with different letters in the same column indicate statistically significant differences in the antioxidant activity as function of the extraction method measured by the same technique (*p <* 0.05).

## Data Availability

The data presented in this article are available upon reasonable request, from the corresponding author.

## Supplementary Materials

The following are available online at https://www.mdpi.com/article/10.3390/foods10092201/s1, material SS.1: Cumulative SC-CO_2_ extraction kinetics of carotenoids from mango at 55 °C and 35 MPa with ethanol and acetone as co-solvents, material SS.2: Extraction yield obtained under the different experimental combinations performed for the optimization process, material SS.3: Statistical analysis of the models applied for the optimized extraction of carotenoids from mango with SC-CO_2_ modified with EtOH or acetone, material SS.4: Carotenoid analysis via UPLC.

## References

[1] Villacís-Chiriboga J., Elst K., Van Camp J., Vera E., Ruales J. (2020). Valorization of byproducts from tropical fruits: Extraction methodologies, applications, environmental, and economic assessment: A review (Part 1: General overview of the byproducts, traditional biorefinery practices, and possible applications). Compr. Rev. Food Sci. Food Saf..

[2] Rodriguez-Concepcion M., Avalos J., Bonet M.L., Boronat A., Gomez-Gomez L., Hornero-Mendez D., Limon M.C., Meléndez-Martínez A.J., Olmedilla-Alonso B., Palou A. (2018). A global perspective on carotenoids: Metabolism, biotechnology, and benefits for nutrition and health. Prog. Lipid Res..

[3] Zhang H., Qi R., Mine Y. (2019). The impact of oolong and black tea polyphenols on human health. Food Biosci..

[4] Arbizu-Berrocal S.H., Kim H., Fang C., Krenek K.A., Talcott S.T., Mertens-Talcott S.U. (2019). Polyphenols from mango (*Mangifera indica* L.) modulate PI3K/AKT/mTOR-associated micro-RNAs and reduce inflammation in non-cancer and induce cell death in breast cancer cells. J. Funct. Foods.

[5] Servaes K., Maesen M., Prandi B., Sforza S., Elst K. (2015). Polar Lipid Profile of Nannochloropsis oculata Determined Using a Variety of Lipid Extraction Procedures. J. Agric. Food Chem..

[6] Molino A., Mehariya S., di Sanzo G., Larocca V., Martino M., Leone G.P., Marino T., Chianese S., Balducchi R., Musmarra D. (2020). Recent developments in supercritical fluid extraction of bioactive compounds from microalgae: Role of key parameters, technological achievements and challenges. J. CO_2_ Util..

[7] de Andrade Lima M., Kestekoglou I., Charalampopoulos D., Chatzifragkou A. (2019). Supercritical Fluid Extraction of Carotenoids from Vegetable Waste Matrices. Molecules.

[8] Garcia-Mendoza M.P., Paula J.T., Paviani L.C., Cabral F.A., Martinez-Correa H.A. (2015). Extracts from mango peel by-product obtained by supercritical CO_2_ and pressurized solvent processes. LWT Food Sci. Technol..

[9] Sánchez-Camargo A.d.P., Gutiérrez L.F., Vargas S.M., Martinez-Correa H.A., Parada-Alfonso F., Narváez-Cuenca C.E. (2019). Valorisation of mango peel: Proximate composition, supercritical fluid extraction of carotenoids, and application as an antioxidant additive for an edible oil. J. Supercrit. Fluids.

[10] Babbar N., Dejonghe W., Sforza S., Elst K. (2017). Enzymatic pectic oligosaccharides (POS) production from sugar beet pulp using response surface methodology. J. Food Sci. Technol..

[11] Elst K., Maesen M., Jacobs G., Bastiaens L., Voorspoels S., Servaes K. (2018). Supercritical CO_2_ Extraction of Nannochloropsis sp.: A Lipidomic Study on the Influence of Pretreatment on Yield and Composition. Molecules.

[12] Lichtenthaler H.K., Buschmann C. (2001). Chlorophylls and Carotenoids: Measurement and Characterization by UV-VIS Spectroscopy. Curr. Protoc. Food Anal. Chem..

[13] Bijttebier S.K.A., D’Hondt E., Hermans N., Apers S., Voorspoels S. (2013). Unravelling ionization and fragmentation pathways of carotenoids using orbitrap technology: A first step towards identification of unknowns. J. Mass Spectrom..

[14] De Paepe D., Servaes K., Noten B., Diels L., De Loose M., Van Droogenbroeck B., Voorspoels S. (2013). An improved mass spectrometric method for identification and quantification of phenolic compounds in apple fruits. Food Chem..

[15] Brand-Williams W., Cuvelier M.E., Berset C. (1995). Use of a free radical method to evaluate antioxidant activity. LWT Food Sci. Technol..

[16] Benzie I.F.F., Strain J.J. (1996). The ferric reducing ability of plasma (FRAP) as a measure of “antioxidant power”: The FRAP assay. Anal. Biochem..

[17] Machmudah S., Wahyudiono, Goto M., Fornari T., Stateva R. (2015). Supercritical Fluid Extraction of Carotenoids. High Pressure Fluid Technology for Green Food Processing.

[18] Tirado D.F., Calvo L. (2019). The Hansen theory to choose the best cosolvent for supercritical CO_2_ extraction of Β-carotene from Dunaliella salina. J. Supercrit. Fluids.

[19] Calvo M.M., Dado D., Santa-María G. (2007). Influence of extraction with ethanol or ethyl acetate on the yield of lycopene, β-carotene, phytoene and phytofluene from tomato peel powder. Eur. Food Res. Technol..

[20] Meneses M.A., Caputo G., Scognamiglio M., Reverchon E., Adami R. (2015). Antioxidant phenolic compounds recovery from *Mangifera indica* L. by-products by supercritical antisolvent extraction. J. Food Eng..

[21] Del Valle J.M., Urrego F.A. (2012). Free solute content and solute-matrix interactions affect apparent solubility and apparent solute content in supercritical CO_2_ extractions. A hypothesis paper. J. Supercrit. Fluids.

[22] Machmudah S., Shotipruk A., Goto M., Sasaki M., Hirose T. (2006). Extraction of astaxanthin from Haematococcus pluvialis using supercritical CO_2_ and ethanol as entrainer. Ind. Eng. Chem. Res..

[23] Volf I., Ignat I., Neamtu M., Popa V.I. (2014). Thermal stability, antioxidant activity, and photo-oxidation of natural polyphenols. Chem. Pap..

[24] Sato T., Ikeya Y., Adachi S.I., Yagasaki K., Nihei K.I., Itoh N. (2019). Extraction of strawberry leaves with supercritical carbon dioxide and entrainers: Antioxidant capacity, total phenolic content, and inhibitory effect on uric acid production of the extract. Food Bioprod. Process..

[25] Schieber A., Ullrich W., Carle R. (2000). Characterization of polyphenols in mango puree concentrate by HPLC with diode array and mass spectrometric detection. Innov. Food Sci. Emerg. Technol..

[26] Agatonovic-Kustrin S., Kustrin E., Morton D.W. (2018). Phenolic acids contribution to antioxidant activities and comparative assessment of phenolic content in mango pulp and peel. S. Afr. J. Bot..

[27] Mercadante A.Z., Rodriguez-Amaya D.B. (1998). Effects of Ripening, Cultivar Differences, and Processing on the Carotenoid Composition of Mango. J. Agric. Food Chem..

[28] Pott I., Marx M., Neidhart S., Mühlbauer W., Carle R. (2003). Quantitative determination of β-carotene stereoisomers in fresh, dried, and solar-dried mangoes (*Mangifera indica* L.). J. Agric. Food Chem..

[29] Liang M., Su X., Yang Z., Deng H., Yang Z., Liang R., Huang J. (2020). Carotenoid composition and expression of carotenogenic genes in the peel and pulp of commercial mango fruit cultivars. Sci. Hortic..

[30] Cuttriss A.J., Cazzonelli C.I., Wurtzel E.T., Pogson B.J., Rébeillé F., Douce R. (2011). Carotenoids. Advances in Botanical Research.

[31] Haque S., Begum P., Khatun M., Islam S.N. (2015). Total Carotenoid Content in Some Mango (*Mangifera indica*) Varieties of Bangladesh. Int. J. Pharm. Sci. Res..

[32] Mercado-Mercado G., Montalvo-González E., González-Aguilar G.A., Alvarez-Parrilla E., Sáyago-Ayerdi S.G. (2018). Ultrasound-assisted extraction of carotenoids from mango (*Mangifera indica* L. ‘Ataulfo’) by-products on in vitro bioaccessibility. Food Biosci..

[33] de Andrade Lima M., Charalampopoulos D., Chatzifragkou A. (2018). Optimisation and modelling of supercritical CO_2_ extraction process of carotenoids from carrot peels. J. Supercrit. Fluids.

[34] Vithana M.D.K., Singh Z., Johnson S.K. (2019). Harvest maturity stage affects the concentrations of health-promoting compounds: Lupeol, mangiferin and phenolic acids in the pulp and peel of ripe ‘Kensington Pride’ mango fruit. Sci. Hortic..

[35] Yao L., Fan L., Duan Z. (2020). Effect of different pretreatments followed by hot-air and far-infrared drying on the bioactive compounds, physicochemical property and microstructure of mango slices. Food Chem..

[36] Rumainum I.M., Worarad K., Srilaong V., Yamane K. (2018). Fruit quality and antioxidant capacity of six Thai mango cultivars. Agric. Nat. Resour..

[37] Pleguezuelos-Villa M., Nácher A., Hernández M.J., Ofelia Vila Buso M.A., Ruiz Sauri A., Díez-Sales O. (2019). Mangiferin nanoemulsions in treatment of inflammatory disorders and skin regeneration. Int. J. Pharm..

[38] Silva L.d.O., Ranquine L.G., Monteiro M., Torres A.G. (2019). Pomegranate (*Punica granatum* L.) seed oil enriched with conjugated linolenic acid (cLnA), phenolic compounds and tocopherols: Improved extraction of a specialty oil by supercritical CO_2_. J. Supercrit. Fluids.

[39] Liu Z., Wu H.L., Xie L.X., Hu Y., Fang H., Sun X.D., Wang T., Xiao R., Yu R.Q. (2017). Direct and interference-free determination of thirteen phenolic compounds in red wines using a chemometrics-assisted HPLC-DAD strategy for authentication of vintage year. Anal. Methods.

[40] Villacís-Chiriboga J., García-Ruiz A., Baenas N., Moreno D.A., Meléndez-Martínez A.J., Stinco C.M., Jerves-Andrade L., León-Tamariz F., Ortiz-Ulloa J., Ruales J. (2018). Changes in phytochemical composition, bioactivity and in vitro digestibility of guayusa leaves (Ilex guayusa Loes.) in different ripening stages. J. Sci. Food Agric..

[41] Huang C.Y., Kuo C.H., Wu C.H., Kuan A.W., Guo H.R., Lin Y.H., Wang P.K. (2018). Free Radical-Scavenging, Anti-Inflammatory, and Antibacterial Activities of Water and Ethanol Extracts Prepared from Compressional-Puffing Pretreated Mango (*Mangifera indica* L.) Peels. J. Food Qual..

[42] Ma X., Wu H., Liu L., Yao Q., Wang S., Zhan R., Xing S., Zhou Y. (2011). Polyphenolic compounds and antioxidant properties in mango fruits. Sci. Hortic..

[43] Fratianni A., Adiletta G., Di Matteo M., Panfili G., Niro S., Gentile C., Farina V., Cinquanta L., Corona O. (2020). Evolution of carotenoid content, antioxidant activity and volatiles compounds in dried mango fruits (*Mangifera indica* L.). Foods.

[44] Jiménez-Escrig A., Jiménez-Jiménez I., Sánchez-Moreno C., Saura-Calixto F. (2000). Evaluation of free radical scavenging of dietary carotenoids by the stable radical 2,2-diphenyl-1-picrylhydrazyl. J. Sci. Food Agric..

[45] Quan T.H., Benjakul S., Sae-leaw T., Balange A.K., Maqsood S. (2019). Protein–polyphenol conjugates: Antioxidant property, functionalities and their applications. Trends Food Sci. Technol..

