# Supplementary material for: Supercritical CO2 Extraction of Bioactive Compounds from Mango (Mangifera indica L.) Peel and Pulp
Source: Foods. 2021 Sep 17;10(9):2201. doi: 10.3390/foods10092201 (PMC8464866; doi:10.3390/foods10092201)
Supplement: Supplementary file 1 [file foods-10-02201-s001.zip › foods-1354830-supplementary.pdf]

Supercritical CO_2 extraction of bioactive compounds from mango (*Mangifera indica* L.) peel and pulp

José Villacís-Chiriboga[1, 2, 3], Stefan Voorspoels[1], Maarten Uyttebroek[1], Jenny Ruales[3], John Van Camp[2], Edwin Vera[3], Kathy Elst[1*]

Supplementary material

Supplementary material SS.1

Cumulative SC-CO_2 extraction kinetics of carotenoids from mango at 55 °C and 35 MPa with ethanol and acetone as co-solvents

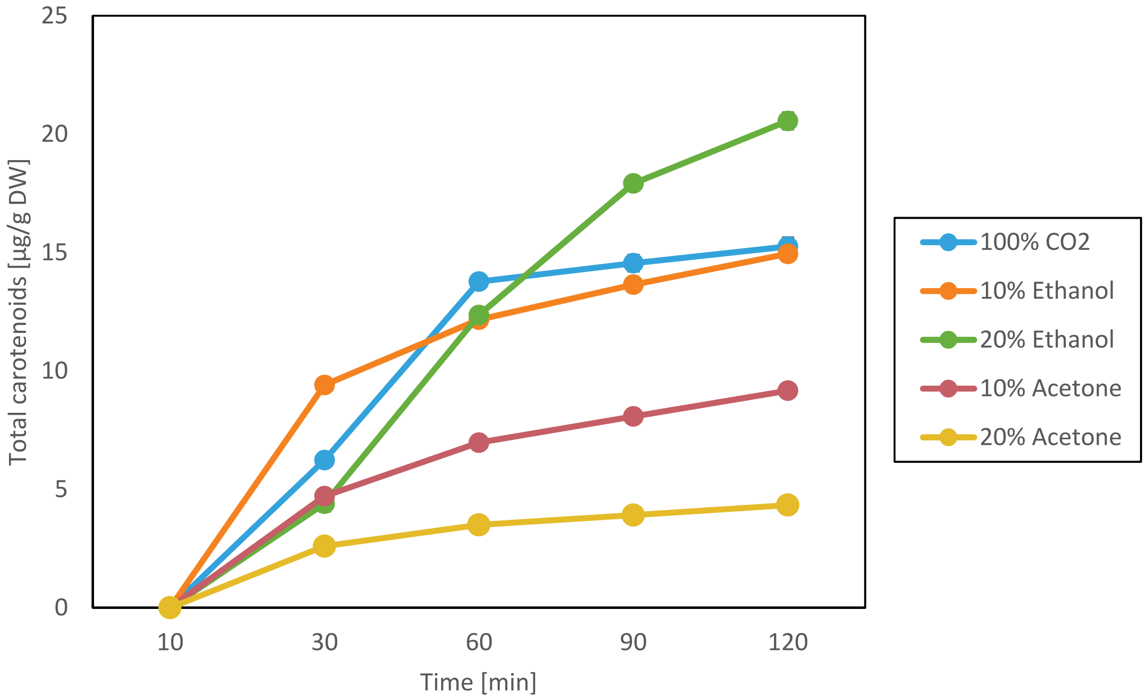

Supplementary material SS.2

Extraction yield obtained under the different experimental combinations performed for the optimization process.

	**35 MPa**	**10 MPa**
35 °C; 100% CO_2	12.81 ± 0.22^{ef}	0.59 ± 0.04^{e}
35 °C; 10% EtOH	10.10 ± 0.30^{d}	0.36 ± 0.02^{abcd}
35 °C; 20% EtOH	16.66 ± 0.40^{i}	3.17 ± 0.00^{g}
35 °C; 10% Acet	12.52 ± 0.31^{e}	0.47 ± 0.00^{de}
35 °C; 20% Acet	13.22 ± 0.30f^{g}	0.21 ± 0.00^{a}
55 °C; 100% CO_2	15.26 ± 0.41^{h}	0.21 ± 0.00^{a}
55 °C; 10% EtOH	14.95 ± 0.10^{h}	2.00 ± 0.02^{f}
55 °C; 20% EtOH	20.56 ± 0.39^{j}	4.70 ± 0.35^{h}
55 °C; 10% Acet	9.17 ± 0.02^{c}	0.35 ± 0.04^{abcd}
55 °C; 20% Acet	4.33 ± 0.01^{a}	0.45 ± 0.03^{cde}
70 °C; 100% CO_2	9.65 ± 0.21^{cd}	0.25 ± 0.01^{ab}
70 °C; 10% EtOH	13.85 ± 1.22^{g}	0.30 ± 0.00^{abc}
70 °C; 20% EtOH	16.94 ± 0.10^{b}	0.44 ± 0.00^{cde}
70 °C; 10% Acet	12.31 ± 0.06^{e}	0.46 ± 0.00^{de}
70 °C; 20% Acet	8.39 ± 0.39^{i}	0.40 ± 0.02^{bcd}

Values are mean ± relative difference (n = 2).

Means with different letters in the same row indicate statistically significant differences between the carotenoids content and the extraction variables

Supplementary material SS.3

Statistical analysis of the models applied for the optimized extraction of carotenoids from mango with SC-CO_2 modified with EtOH or acetone

Extraction performed with ethanol

Source	*Sum of squares*	*DF*	*Medium square*	*F-ratio*	*p-value*
**1:** Temperature [°C]	0,849	1	0,849	0,41	0,525
**2:** Pressure [MPa]	1567,16	1	1567,16	763,35	0
**3:** Ethanol [%]	93,531	1	93,531	45,56	0
Temperature square	51,563	1	51,563	25,12	0
Pressure × co-solvent	14,087	1	14,087	6,86	0,014
Co-solvent square	18,275	1	18,275	8,9	0,006
Blocks	0,032	1	0,032	0,02	0,901
Total error	57,484	28	2,053		
Total (corr.)	1802,24	35			

R^2 = 96,81
R^2 adj = 96,013

Extraction performed with acetone

Source	*Sum of squares*	*DF*	*Medium square*	*F-ratio*	*p-value*
**1:** Temperature [°C]	13,161	1	13,161	3,70	0,064
**2:** Pressure [MPa]	996,88	1	996,88	280,22	0,000
**3:** Acetone [%]	23,027	1	23,027	6,47	0,017
Temperature × Pressure	12,166	1	12,166	3,42	0,075
Pressure × Co-solvent	23,111	1	23,111	6,50	0,016
Blocks	0,002	1	0,002	0,00	0,983
Total error	103,167	29	3,557		
Total (corr.)	1162,1	35			

R^2 = 91,122
R^2 adj = 89,286

Supplementary material SS.4

Chromatograms obtained on the analysis for carotenoids and phenolics.

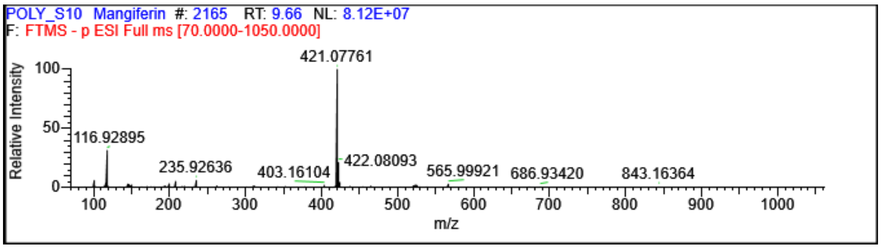

Fig 1. Phenolic compounds analysis via UPLC

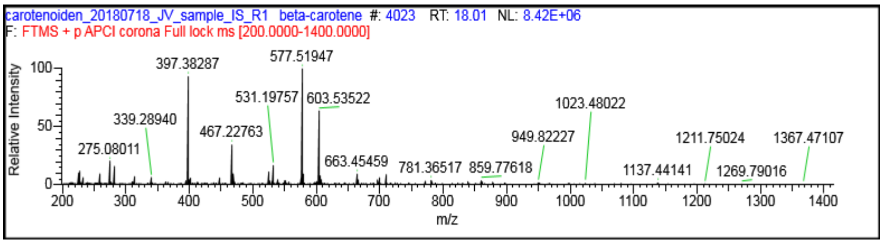

Fig 2. Carotenoid analysis via UPLC
